# Proteomics data analysis using multiple statistical approaches identified proteins and metabolic networks associated with sucrose accumulation in sugarcane

**DOI:** 10.1186/s12864-022-08768-2

**Published:** 2022-07-22

**Authors:** Ao-Mei Li, Zhong-Liang Chen, Cui-Xian Qin, Zi-Tong Li, Fen Liao, Ming-Qiao Wang, Prakash Lakshmanan, Yang-Rui Li, Miao Wang, You-Qiang Pan, Dong-Liang Huang

**Affiliations:** 1 Key Laboratory of Sugarcane Biotechnology and Genetic Improvement (Guangxi), Ministry of Agriculture and Rural Affairs, Guangxi Key Laboratory of Sugarcane Genetic Improvement /Sugarcane Research Institute, Guangxi Academy of Agricultural Sciences, 530007 Nanning, China; 2 Melbourne Integrative Genomics and School of Mathematics and Statistics, the University of Melbourne, 3010 Parkville, VIC Australia; 3 Abmart, 200033 Shanghai, China; 4 Interdisciplinary Research Center for Agriculture Green Development in Yangtze River Basin, College of Resources and Environment, Southwest University, 400716 Chongqing, China; 5 Queensland Alliance for Agriculture and Food Innovation, University of Queensland, 4067 St Lucia, QLD Australia

**Keywords:** Sugarcane, Proteomics, Differentially abundant protein, Statistical approach, Sucrose accumulation

## Abstract

**Background:**

Sugarcane is the most important sugar crop, contributing > 80% of global sugar production. High sucrose content is a key target of sugarcane breeding, yet sucrose improvement in sugarcane remains extremely slow for decades. Molecular breeding has the potential to break through the genetic bottleneck of sucrose improvement. Dissecting the molecular mechanism(s) and identifying the key genetic elements controlling sucrose accumulation will accelerate sucrose improvement by molecular breeding. In our previous work, a proteomics dataset based on 12 independent samples from high- and low-sugar genotypes treated with ethephon or water was established. However, in that study, employing conventional analysis, only 25 proteins involved in sugar metabolism were identified .

**Results:**

In this work, the proteomics dataset used in our previous study was reanalyzed by three different statistical approaches, which include a logistic marginal regression, a penalized multiple logistic regression named Elastic net, as well as a Bayesian multiple logistic regression method named Stochastic search variable selection (SSVS) to identify more sugar metabolism-associated proteins. A total of 507 differentially abundant proteins (DAPs) were identified from this dataset, with 5 of them were validated by western blot. Among the DAPs, 49 proteins were found to participate in sugar metabolism-related processes including photosynthesis, carbon fixation as well as carbon, amino sugar, nucleotide sugar, starch and sucrose metabolism. Based on our studies, a putative network of key proteins regulating sucrose accumulation in sugarcane is proposed, with glucose-6-phosphate isomerase, 2-phospho-D-glycerate hydrolyase, malate dehydrogenase and phospho-glycerate kinase, as hub proteins.

**Conclusions:**

The sugar metabolism-related proteins identified in this work are potential candidates for sucrose improvement by molecular breeding. Further, this work provides an alternative solution for omics data processing.

**Supplementary Information:**

The online version contains supplementary material available at 10.1186/s12864-022-08768-2.

## Introduction

Omics data analysis applies statistical and computational methodology to determine the key genes, proteins and metabolites that significantly regulate various plant growth and developmental processes [1, 2]. A major challenge is that the omics data usually comprise thousands or even millions of features, but the sample size used can be relatively limited to detect potentially biologically-relevant proteins or its attributes. This creates the so-called p > > n problem [3], i.e., the number of features is massively larger than the number of samples needed to make reliable findings. With large high dimensional datasets, classical statistical models such as linear regression or logistic regression become oversaturated and ineffective, and thus cannot provide useful outcomes.

One strategy to address this analytical limitation is to apply a marginal regression approach or a *t*-test approach to analyse one bio-feature at a time. Such an approach can often identify a set of hundreds of significant features [4]. Alternatively, another recently proposed approach is penalized regression [4], which analyse multiple features simultaneously, and use a penalty function to shrink the effects of unimportant features towards zero to exclude them to keep only the important ones in the model. The penalized regression approach has also been suggested as a tool to estimate the sparse inverse covariation matrix, which can be applied to construct gene or protein interaction networks [5]. A third option is to use Bayesian regression methods. The Bayesian approaches are in fact closely connected to the penalised regression, in the sense that they interpret the penalty function as a prior distribution, and then combine the prior and the model likelihood to form a posterior distribution. Then simulation-based computational methods such as Markov Chain Monte Carlo can be used to approximate the posterior distribution. One advantage of the Bayesian regression approaches is that they can directly provide uncertainty measurements such as credible intervals or inclusion probabilities to the regression coefficients, which can be used for inference. Although these newer penalized regression and Bayesian approaches have been proposed to detect association between traits of interest and different kind of omics data, including proteomics, biologists tend to keep on using conventional two sample t-test or simple logistic regression, due to the fact that there is little efforts to illustrate the power and advantages of using the high multiple regression methods for large biological datasets [6].

Sugarcane has the capacity to store sucrose in the stem as the main reserve food [7], and it contributes to > 80% of the world’s sucrose production [8]. High sucrose content is a main target of sugarcane breeding. However, the sucrose content in sugarcane remained plateaued for several decades despite sustained conventional breeding efforts for a long time. Recent success of molecular breeding in other crops suggests its potential for sucrose improvement in sugarcane [9–11]. However, little success has been made so far in relation to identifying key genetic elements that control sucrose accumulation, which will help develop molecular breeding strategies for sugarcane. Some previous studies in sugarcane have identified candidate genes likely to be involved in sugar accumulation, but the molecular mechanism sugar accumulation in this crop remains unknown [12–16].

Ethylene is an effective chemical ripener used in commercial sugarcane crops [17]. In our previous work, application of ethephon (an ethylene releasing compound) in a high- and a low-sugar genotype elicited differential sucrose accumulation responses, with low-sugar variety accumulating relatively more sugar than the high-sugar clone [18]. Using this system, proved to be an effective model for dissecting the molecular regulation of sugar accumulation in sugarcane, we established a proteomics dataset representing early stages of sugar accumulation in both clones in response to ethephon treatment. The dataset was based on 12 independent samples and had about 3,000 protein features. Differentially abundant proteins (DAPs) were identified using Mann-Whitney Test with a significance level of p < 0.05 adjusted by Benjamini-Hochberg Correction and fold change over 1.2. But, in this analysis only 25 proteins related to sugar metabolism were identified [19]. To determine whether our previous analysis detected only a fraction of proteins associated with sugar accumulation, we evaluated more sophisticated and powerful methods for analysis. Here we used three different statistical approaches, namely, the marginal logistic regression method [20], a logistic penalized regression approach named Elastic net method [21], and a logistic Bayesian stochastic search variable selection (SSVS) method [22] to re-analyse the proteomics dataset to determine the most effective analytical methods for proteomics data with < 20 sample size and to maximise the extraction of valuable information for the study.

## Materials and methods

### Sample collection and proteomics data set preparation

Plant growth conditions, sample collection and proteomics dataset establishment used for this study are described in our previous work [19]. Briefly, two sugarcane genotypes, ROC22, a high-sugar (on average 15% sucrose content, ROC5 × ROC69-46), and GT86-877, a low-sugar (on average 6% sucrose content, GT82-10 × GT73-11) genotype were used for the experiment. Plants were grown in the experimental farm of Sugarcane Research Institute, Guangxi Academy of Agricultural Sciences, Nanning, Guangxi China in 2016. and they were treated with deionized water or 400 mg/L Ethephon solution as foliar spray till run-off from the lamina in early sucrose accumulation stage (8 months old crop), in mid-October. Developing stalk tissues, 20 cm above the node attached to the second youngest fully expanded leaf, which is highly photosynthetically active, were sampled on day 7 following ethylene treatment. Pooled sample of tissues collected from six individual plants from each replicate plot constitute a single biological replicate. Three biological replicates were collected for each clone from each treatment and they were flash-frozen in liquid nitrogen for proteome analysis by iTRAQ method.

### Batch effect correction

The following linear mixed model (LMM) was applied to correct the potential batch effect caused by the data sampling process (i.e., plates in MS):

1 $$\log-{\mathrm{inten}}_{ijk}=g_ja_i+b_j+s_{jk}+e_{ijk},$$

where log-inten_*ijk*_ represents the intensity value at the protein features *k* of the replicate *i* within the group j, *g*_*i*_ is the design matrix of the (fixed) group effect and *a*_*j*_ is the corresponding regression coefficients, *b*_*j*_ is the random effect of the replicate *j*, *s*_*jk*_ is the (interactive) random effect of the protein *k* and replicate *j*, and *e*_*ijk*_ is the model residual. The random effects *r*_*j*_. *s*_*jk*_ and the residuals *e*_*ijk*_ are assumed to mutually follow normal distributions $N(0,\sigma _{r}^{2})$, $N(0,\sigma _{s}^{2})$, and $N(0,\sigma _{e}^{2})$, respectively. The regression parameters of LMM was estimated by the maximum likelihood method, implemented by the R package (version 4.0.3) lme4.

Principal component analysis (PCA) was conducted by the R function “prcomp” on the proteomics data to visualize the structure and variation among the samples, and check with the performance of batch effect correction. The residuals.

2 $$x_{ik}=\;\log-{\mathrm{inten}}_{ijk}-s_{jk}$$

were considered as features being used in the follow-up statistical analyses.

### Differential protein abundance analysis

The marginal regression analyzed one spectrum at a time, defined as.

3 $$x_{ik}=\beta_0\:+\:y_i\beta_k+e_{ik},$$

where *x*_*ik*_ is the (adjusted) intensity of the proteins *k* at the sample *i*, *y*_*i*_ is the binary group indicator, ***z***_*i*_ is the design matrix of the covariate variables, *β*_0_ is the model intercept, and *β*_*k*_ is the effect of protein features *k* which is of primary interest, and *e*_*ik*_ is the residual error which is assumed to follow a normal distribution.

A *t*-test was conducted on the regression coefficient *β*_*k*_, and p-value for the proteins was calculated. The *p*-values were adjusted by the conservative Bonferroni adjustment to control the multiplicity.

Additionally, a penalized logistic regression approach named Elastic net [21] was applied to simultaneously analyze all the protein features:

4 $$\begin{gathered} \mathop {\hbox{min} }\limits_{{({\mathbf{\alpha }},{\beta _k})}} \sum\limits_{{i=1}}^{n} {[{y_i}\log {\pi _i}+(1 - {y_i})\log (1 - {\pi _i})]+\lambda } [\omega \sum\limits_{{k=1}}^{p} {\left| {{\beta _k}} \right|} +(1 - \omega )\sum\limits_{{k=1}}^{p} {\beta _{k}^{2}]} , \hfill \\ \hfill \\ \end{gathered}$$

where ${\pi _i}=\frac{{\exp ({\beta _0}+\sum\limits_{{k=1}}^{p} {{x_{ik}}{\beta _k}} )}}{{1+\exp ({\beta _0}+\sum\limits_{{k=1}}^{p} {{x_{ik}}{\beta _k}} )}}$. Variables *y*_*i*_ and *x*_*ik*_, and regression parameters *β*_*0*_ and *β*_*k*_ are defined as the way as in Eq. (3), but the difference is that now in (4) the multiple protein variables are simultaneously analyzed in the same model. Another major difference from the simple regression model (3) is that in (4) the group variable *y*_*i*_ is treated as binary response variables, while the spectra *x*_*ik*_ are treated as explanatory variables. In (4), a penalty term combining the *l*_1_ and *l*_2_ norm penalties: $\lambda [\omega \sum\limits_{{k=1}}^{p} {\left| {{\beta _k}} \right|} +(1 - \omega )\sum\limits_{{k=1}}^{p} {\beta _{k}^{2}]}$ was introduced to shrink the effects of un-important features (i.e., a protein which is not associated with the group variable) to be zero, and only keep the important proteins into the model. The tuning parameter *λ* (*λ* > 0) determines the degree of shrinkage of the regression parameters, and how many proteins should be included into the model, while the weight parameter ω (0 < ω < 1) determines the relative importance of the *l*_1_ and *l*_2_ penalties. The advantage of using such a mixture of *l*_1_ and *l*_2_ penalties over using merely the *l*_1_ penalty (which leads to theLASSO regression) is that the mixture penalty could account for the dependency structure among different features, and simultaneously select multiple correlated features into the model. The optimal values of *λ* and ω are determined by nested cross-validation. The proteins having non-zero regression coefficients were considered to have significant features. The numerical estimation of model (4) was conducted by the R package glmnet (version 4.0).

One disadvantage of the logistic Elastic net approach is that the uncertainty measures of the regression coefficients are not available, especially when the sample size is limited. Therefore, no multiple testing procedure could be applied to control the false positives. This motivates us to use an alternative approach named Bayesian stochastic search selection. Unlike Elastic net (4) which adds the combination of *l*_1_ and *l*_2_ penalty of the regression parameters to the log-likelihood function, in SSVS, the regression coefficient is assumed to follow the so-called spike and slab prior distribution as:

5 $$P({\beta _j}|{r_j}) \propto (1 - {r_j}){{\text{I}}_{\{ {\beta _j}=0\} }}+{r_j}N(0,\sigma _{j}^{2}),$$

The prior is a mixture of normal distribution with zero mean and unknown variance $\sigma _{j}^{2}$ and point mass at zero. The binary indicator variable *r*_*j*_ determines whether the regression parameter is included into the model. The indicator *r*_*j*_ was further assigned with a Bernouli prior:

$$P\left(r_j\left|w\right.\right)=w^{rj}\left(1-w\right)^{1-rj}$$

and the hyper-parameter ω was specified to a small value, following the assumption that only a small proportion of the spectra was associated with the treatment effects. In this work, we use ω = 0.05.

The variance $\sigma _{j}^{2}$was assigned with an Inverse Gamma prior:

$$P(\sigma_j^2\vert a,b)=\text{Inv}-\text{Gamma}(a,b),$$

where the hyper-parameters were specified as a = b = 0.1, which is non-informative.

Combining these prior distributions and the model likelihood leads to a posterior distribution of the model parameters ${\mathbf{\theta }}=({\beta _j},{r_j},\sigma _{j}^{2},\omega )$:

6 $$P({\mathbf{\theta }}|{\mathbf{y}}) \propto P({\mathbf{y}}|{\mathbf{\theta }})P({\mathbf{\theta }})$$.

Bayesian estimation of a logistic regression is generally recognized as a challenging problem. To ease the computation, the posterior was first decomposed into the following two separate problems [23]:

7 $$\begin{gathered} \phi |{\beta _j}\sim {\text{PG}}({n_i},{v_i}) \hfill \\ {\beta _j}|{y_i},\phi \sim N({v_i}|\sum\limits_{{j=1}}^{p} {{x_{ij}}{\beta _j},1} )P({\beta _j}|{\gamma _j})p({\gamma _j}|w) \hfill \\ \end{gathered}$$

where PG (*n*_*i*_, *v*_*i*_) represents a Polya Gamma distribution. Now the posterior has a hierarchical structure with the second layer equivalent to a normal SSVS regression instead of a logistic SSVS regression. An efficient Gibbs sampler could be used for parameter estimation.

The empirical marginal posterior distribution of the selection indictor: $\hat {P}({\gamma _j}=1|{\mathbf{y}})$ which is often viewed as a posterior inclusion probability (PIP) [24] can be used for the posterior inference. Intuitively, one could use PIP > 0.5 as a criterion to claim the corresponding features to be significant. However, such a heuristic criterion does not necessarily guarantee that the false positives due to simultaneously tests of multiple hypotheses could be effectively controlled [25]. Alternatively, we could also utilize the marginal probability of a feature not being selected: $\hat {P}({\gamma _j}=0|{\mathbf{y}})=1 - \hat {P}({\gamma _j}=1|{\mathbf{y}})$, which is interpreted as a local false discovery rate (LFDR) [26–28]. Based on LFDR, a global level Bayesian FDR (BFDR) [29, 30] could be constructed by combining LFDRs for a group of features. To eliminate the multiplicity, the BFDR should be controlled under a defined threshold, here we specified $\alpha =0.05$guaranteeing that the overall FDR among the multiple hypotheses is less than 0.05. In more detail, the BFDR is calculated as follows. First, the LFDRs for each feature are sorted in ascending order. The average value of the LFDRs for the first *T* features (*T* = 1, … ,*p*) is defined as a BFDR for feature markers. We find the highest possible value of BFDR (the average of the *T* smallest LFDRs), which is still smaller than the given threshold, and the corresponding features are significant.

### KEGG analysis

Pathway analysis was processed by KOBAS (http://kobas.cbi.pku.edu.cn/) against *Zea mays* [31]. The KEGG database, which is used in the KEGG analysis, is developed by Kanehisa Laboratories [32–34]. Hypergeometric test and Fisher’s exact test were used, and the FDR correction method was Benjamini and Hochberg.

### Protein validation by western blot

To verify the DAPs detected by statistical methods are indeed biologically relevant to sugar accumulation, 3 proteins selected randomly from this work and the 2 proteins validated in our previous work [19] were further validated using western blot (WB) based on the protocol we used previously [19]. Briefly, SDS-PAGE was used to separate proteins (20 µg) from each sample. They were then transferred to polyvinylidene fluoride membranes which were incubated with appropriate primary antibodies generated by Abmart and the HRP-conjugated anti-mouse IgG secondary antibody (Abmart, M21001). An enhanced chemiluminescence system (Biouniquer, China) was used to visualize Immune-reactive bands, which were exposed to X-ray film (Kodak). Following this, ImageJ program was used to quantify signal intensities which were normalized to the b-actin signal.

### Protein-protein interaction network construction

Protein-Protein Interaction (PPI) Network Analysis was conducted using STRING (https://string-db.org/) [35]. We selected the gene symbol as input of website https://cn.string-db.org/, chose multiple proteins, used gene symbol as list of names, and selected *Zea mays* as organism. The output was exported in the tsv format. The PPIs in the string-db are based on experimental data or predictions by bioinformatics methods. We mapped the DAPs associated with sucrose accumulation onto the PPI network. The hub genes were determined by the website https://cn.string-db.org/. Nodes with the greatest numbers of interactions with neighboring nodes were considered as hub nodes.

## Results

### Datasets and batch effect correction

**Fig. 1:**
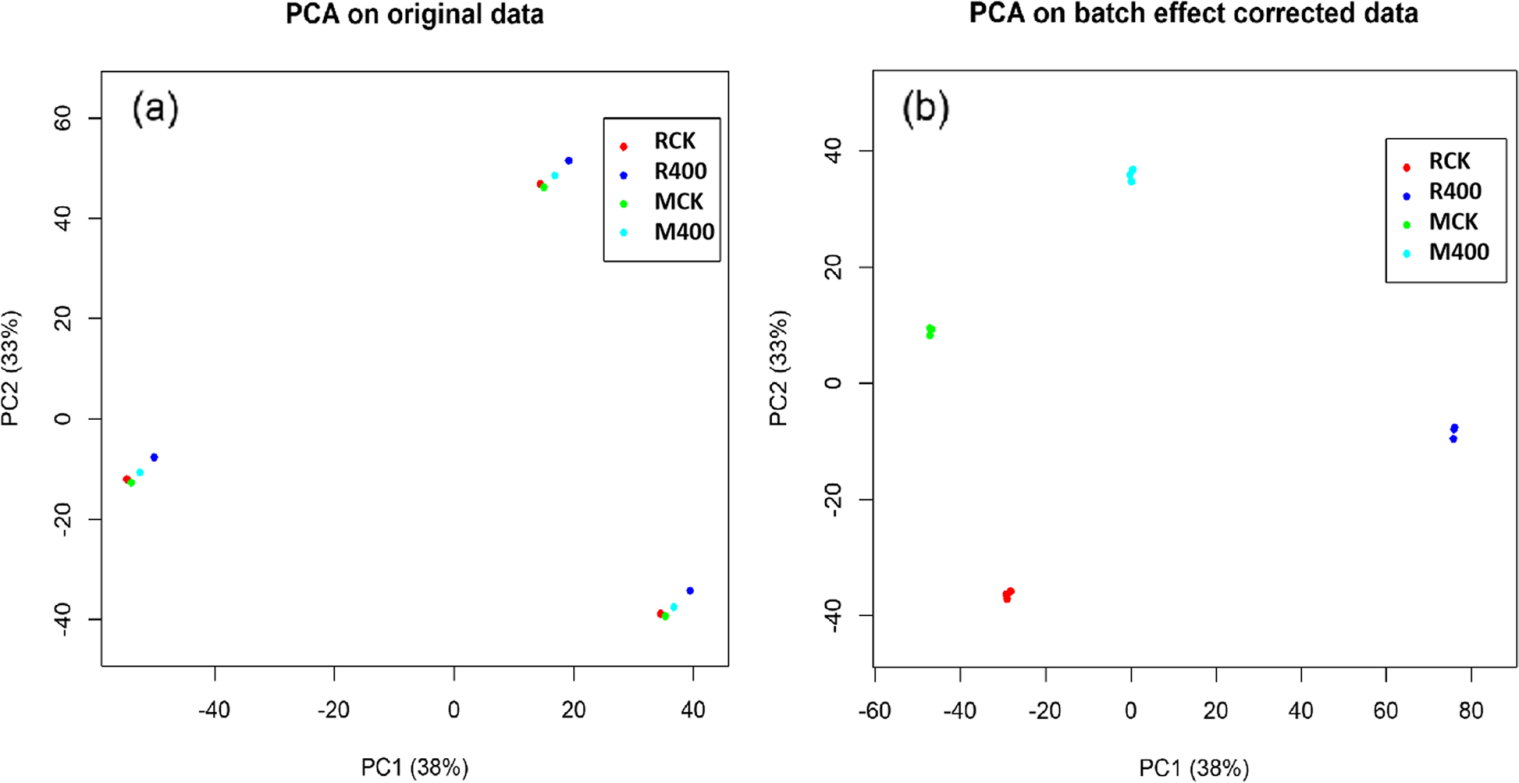
The scatter plot of first and second principal components (PCs) ofsugarcane proteomics data. PCs were calculated from the data before (a) and after (b) batch effect correction

The sucrose content was increased in both genotypes after 3 days of treatment, and it was continued for 7 days [18]. Four samples were collected on 7th day after treatment, and they were labelled as RCK: samples from high-sucrose content sugarcane (on average 15% sucrose content) treated with water; MCK: samples from low-sucrose content sugarcane (on average 6.0% sucrose content) treated with water; R400: samples with high-sucrose content sugarcane treated with 400 mg/L ethephon; and M400: samples from low-sucrose content sugarcane treated with 400 mg/L ethephon. Each group comprises 3 independent biological replicates. By iTRAQ experiment, about 65,000 spectra (19%) were identified from a total of 345,000 spectra of each iTRAQ sample. After data processing, 2,983 proteins were identified [19], and they were included in the statistical analysis. The PCA (Fig. 1a) reveals that the first replicates of the four groups formed one cluster, and the rest of the replicates jointed in the other. The first and second PC explained 81% and 10%, respectively, of the total variation of the spectral data. After batch effect correction, samples from four groups were clearly separated (Fig. 1b). The two PCs explained 41% and 24% of the variation.

### Identification of differentially abundant proteins

The stalk tissue protein abundance in the following treatment comparisons were analysed: (i) high-sugar genotype VS low-sugar genotype (RCK vs. MCK, R400 vs. M400), and (ii) water VS ethephon treatment (MCK vs. M400, RCK vs. R400). Protein abundance variation in different groups were analysed by three different approaches, including a logistic marginal regression, a penalized multiple logistic regression called Elastic net, and a Bayesian multiple logistic regression method namely Stochastic search variable selection (SSVS).

**Fig. 2:**
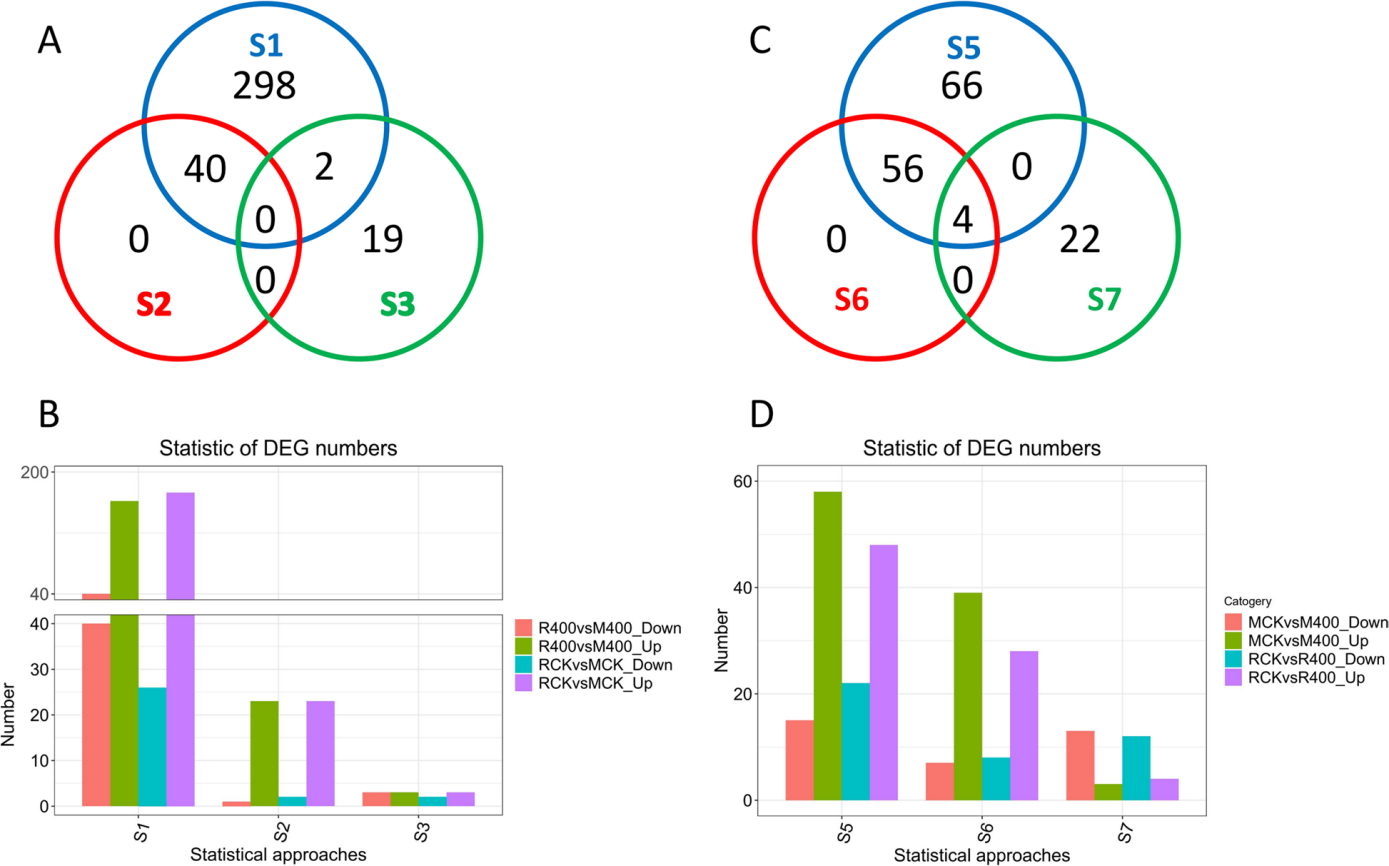
Summary of DAPs detected by three different statistical analysis approaches. Venn diagram of DAPs detected by three methods based on comparisons between genotypes (**A**) and between water and ethephon treatments (**C**). Up-regulated and down-regulated proteins detected in genotype (**B**) and treatments (**D**)comparisons by the three methodsThe protein number of S1-S3 in (**A**) is identical to that in Supplementary Tables 1, 2 and 3, and the number of S5-S7 in (**C**) is identical to that in Supplementary Tables 5, 6 and 7

The simple regression analysis identified 340 proteins that are differentially abundant in the samples with low- and high-sucrose content (RCK vs. MCK, R400 vs. M400) (Table S1). While the multiple regression-based Elastic net detects 40 DAPs (Table S2), which were also detected by the simple regression analysis. The Bayesian SSVS approach identified 21 significantly different DAPs (Table S3), but only 2 of them overlapped with those detected by simple regression method, indicating that 19 new proteins (Table S4) were identified. So, a total of 359 DAPs were detected from the genotype comparison. No common gene was found in the analysis outputs of these three methods (Fig. 2A). When genotypes treated with ethephon were compared (R400 vs. M400), 162, 23 and 3 proteins were found up-regulated in the simple regression, the Elastic net and the Bayesian SSVS analyses, respectively, whereas 40, 1 and 3 proteins, were found to be down-regulated in these three methods, respectively. A similar comparison of genotypes treated with water (RCK vs. MCK), identified 173, 23 and 3 up-regulated and 26, 2 and 2 down-regulated proteins in the simple regression, the Elastic net and the Bayesian SSVS analyses, respectively (Fig. 2B).

In the water and ethephon treatment comparison (MCK vs. M400, RCK vs. R400), the simple regression approach detected 126 DAPs (Table S5), and the Elastic net method identified 60 DAPs (Table S6). Again, all the DAPs identified by Elastic net were also detected by simple regression. However, the Bayesian approach identified 26 DAPs (Table S7), and only 4 of them were common across the Bayesian and the simple regression groups, suggesting that 22 additional DAPs were detected with Bayesian analysis (Table S8). Thus, a total of 148 DAPs were detected from the comparison based on treatments, of which 4 proteins were detected in all the three methods (Fig. 2 C). Three of these proteins were annotated, including enolase (m.4058; m.159,651) and histone H1 (m.136,263). In the comparison of low sugar genotype treated with water and ethephon (MCK vs. M400), 58, 39 and 3 proteins were found up-regulated, and, 15, 7 and 13 proteins were down-regulated in the three methods combined. In a similar comparison between water vs. ethephon treatments in the high-sugar genotype (RCK vs. R400), 48, 28 and 4 up-regulated and 22, 8 and 12 down-regulated proteins were detected between the 3 statistical methods, (Fig. 2D). So, totally 507 DAPs were detected between three methods, of which 306 DAPs were annotated (Table S9).

### Identification of differentially abundant proteins involved in sugar metabolism

**Fig. 3:**
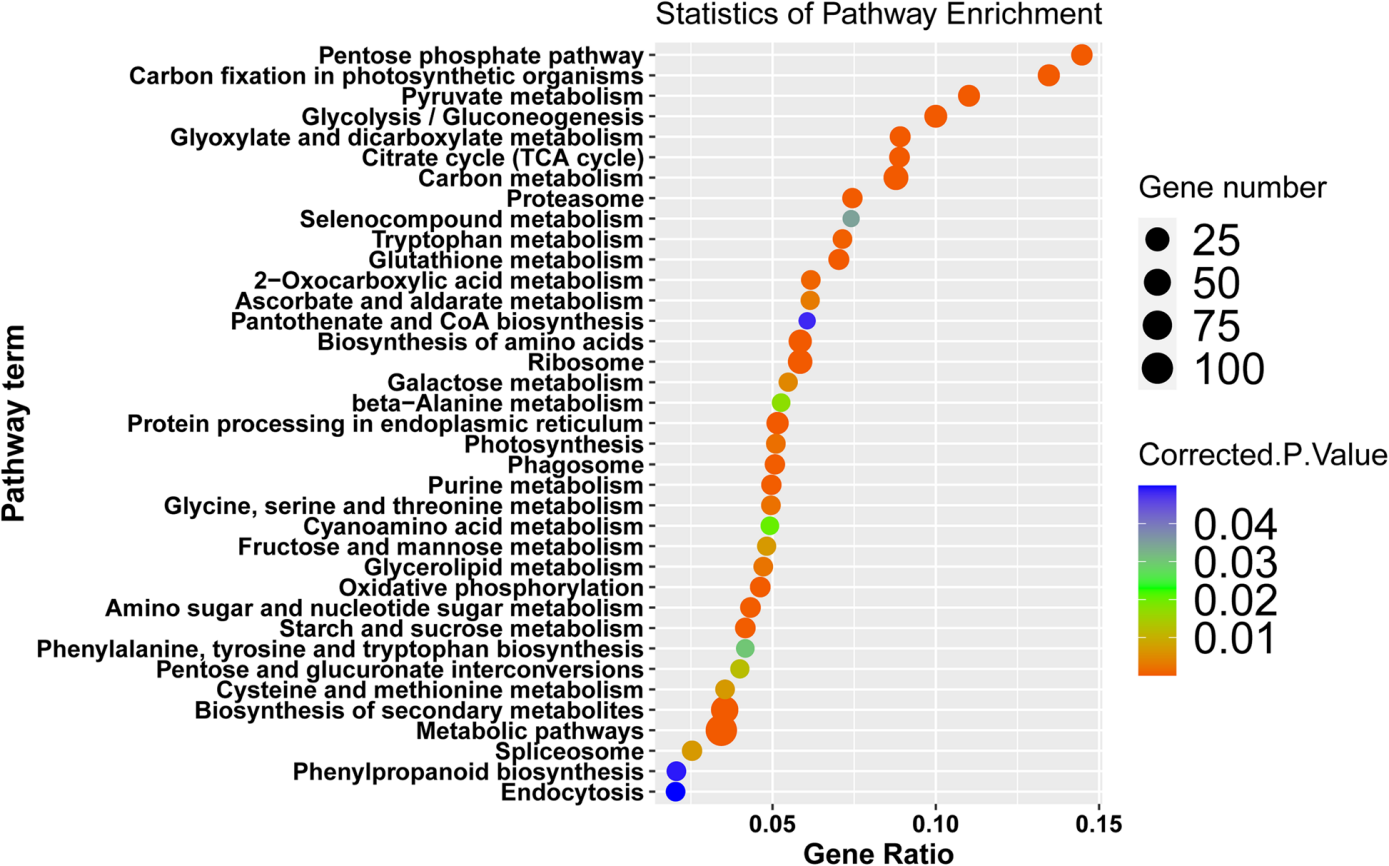
KEGG analysis of annotated DAPs detected by three different statistical approaches. The size of the dots corresponds to the number of DAPs in each pathway. The color displays the significance of enrichment

To find out the DAPs associated with sugar metabolism in sugarcane, KEGG enrichment analysis of 306 DAPs identified by the three methods was conducted (Table S9). Sugar metabolism related pathways, including carbon fixation in photosynthetic organisms, carbon metabolism, photosynthesis, amino sugar and nucleotide sugar metabolism, as well as starch and sucrose metabolism, were enriched (Fig. 3; Table S10). A total of 48 DAPs involved in sugar metabolism were identified from KEGG enrichment analysis (Table S11).

Considering that some proteins associated with sugar metabolism related pathways were not enriched due to their very low abundance, we checked the annotation of all the DAPs detected in this work to find possible missing proteins related to sugar metabolism. We did find one protein m.146,783, annotated as chlorophyll a-b binding protein, which is a photosynthesis - antenna protein, was positively correlated with sugar metabolism in plants. So, the protein m.146,783 is also considered as a candidate target participating in sucrose accumulation in sugarcane. Thus, a total of 49 proteins associated with sugar metabolism were identified in this work (Table 1). Then we compared these 49 DAPs with the DAPs involved in sugar metabolism identified in our previous work [19]. Seven DAPs were found overlapping between two studies, indicating that 42 new DAPs associated with sugar metabolism were identified in this work (Table 1). The mean abundance and standard deviation of those 49 DAP’s are presented in Table S12.

In the photosynthesis pathway, 5 new enzymes, PsaD (m.80,314), oxygen-evolving enhancer (m.150,518/m.77,636), ATP synthase (m.17,250) and ferredoxin reductase (m.27,004) were identified in this work. Similarly, in the carbon fixation pathway, besides PEPC (m.172,065/m.114,523/m.101,304) some other important enzymes such as phosphoenolpyruvate carboxykinase (m.82,036/m.82,032), NADP-dependent malic enzyme (m.96,035/m.58,073), enolase (m.159,644/m.159,651), ribulose-phosphate 3-epimerase (m.37,775) and ribulose-1,5-bisphosphate carboxylase oxygenase (m.120,708) were also identified in this work, compared to our previous work [19]. Interestingly, m.159,651 was the common protein detected by all the three methods, implying an important role for it in the sucrose accumulation process.

While in the carbon metabolism pathway, 23 new proteins were identified in this work, including important enzymes such as, phosphoglycerate kinase (m.138,161), glucose-6-phosphate isomerase (m.101,603), phospho-glucomutase (m.128,432/m.141,239), and 6-phosphogluconate dehydrogenase (m.48,690), etc. In the amino sugar and nucleotide sugar metabolism pathway, 2 new proteins, UDPG pyrophosphorylase (m.25,693) and ADPG pyrophosphorylase (m.85,895) were identified. In the starch and sucrose metabolism pathway, besides the two beta-glucosidases (m.97,819/m.27,280), a glucose-6-phosphate 1-epimerase (m.104,895) and an alpha-glucan phosphorylase (m.45,257) were also detected in this work.

Genes are carriers of genetic information, while proteins execute the gene function. To get moreinsights into the molecular mechanism of sucrose accumulation, we looked for overlaps between the new DAPs and those from our previous work that used the same dataset [19], as well as DEGs reported by Chen et al. [18]. There were 7 overlapping proteins between this work and previous work [19] (Table 1). This analysis also identified genes of 12 DAPs showing differential expression at transcriptional level (Table 1).

**Table 1:**
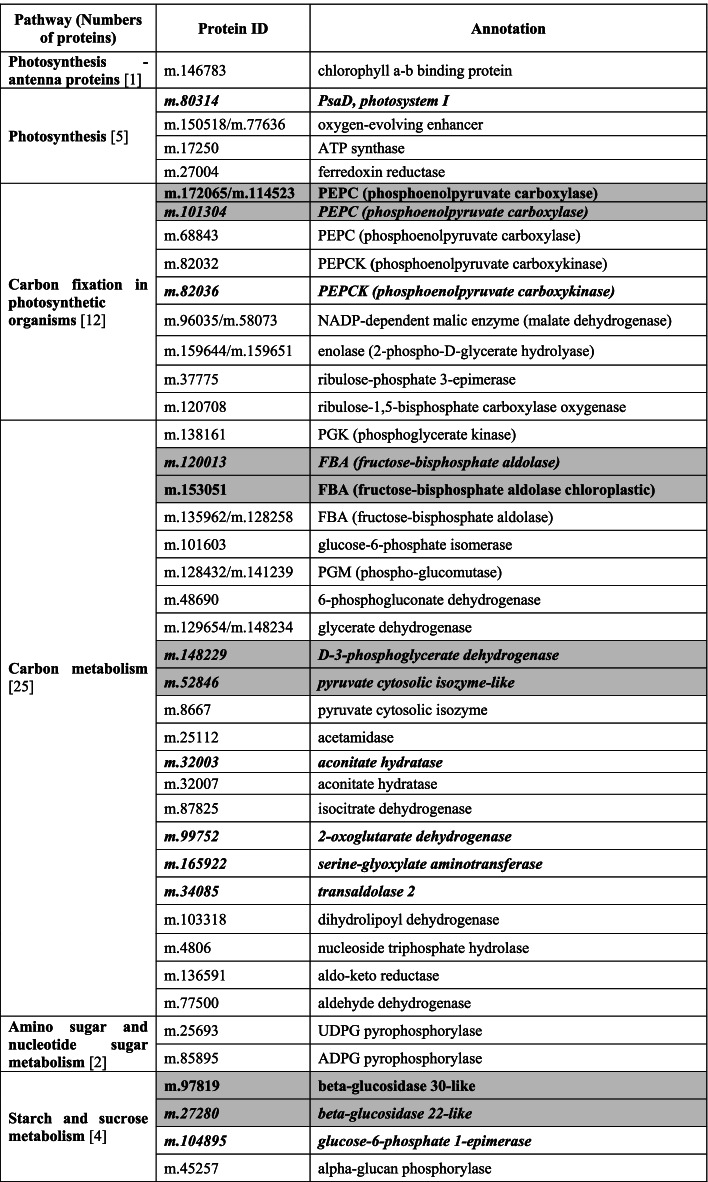
Differentially abundant proteins related to sucrose accumulation identified in this work

Protein ID and names in italic bold indicate DAPs with their encoding genes showing differential expression at transcriptional level [18], and those with bold font with gray background indicate proteins overlapping between those identified in the current and our previous work [19]

### Validation of DAPs by western blot

**Fig. 4:**
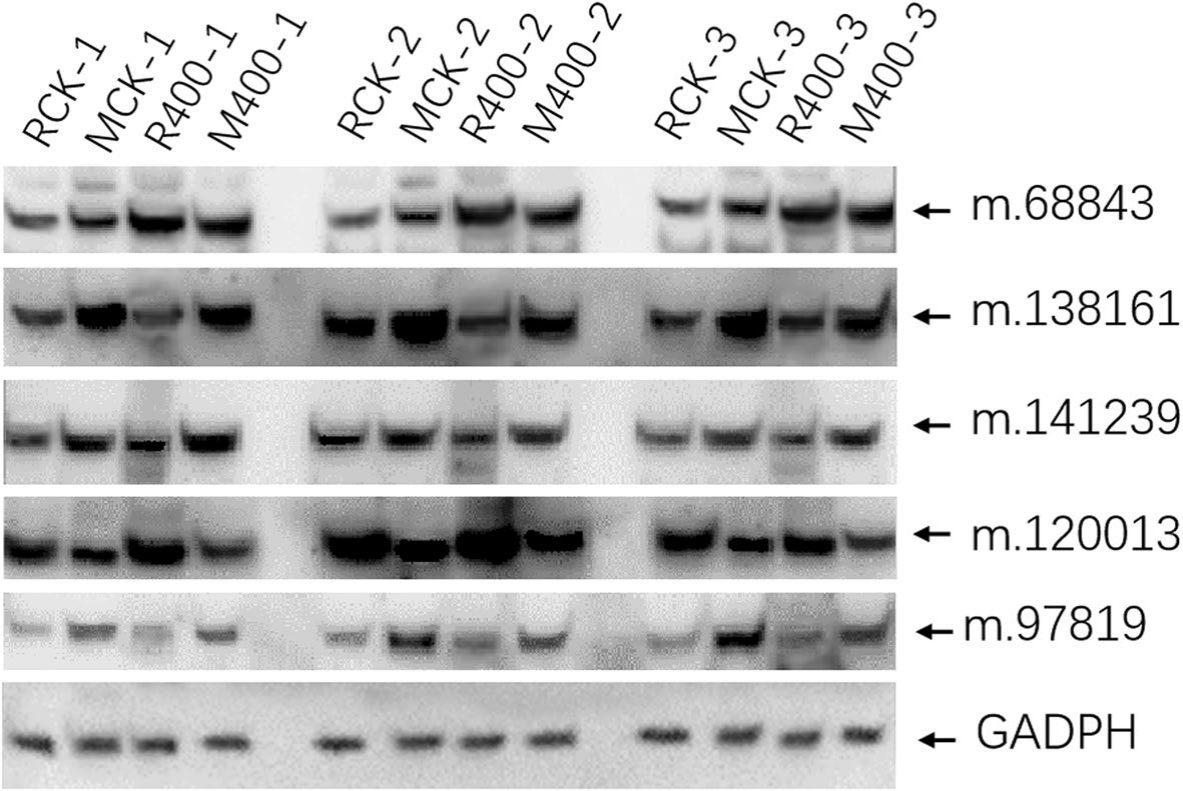
Western blot validation of differentially abundant proteins. RCK: high-sugar genotype with water control; MCK: low-sugar genotype with water control; R400: high-sugar genotype with ethephon treatment; M400: low-sugar genotype with ethephon treatment. Number after sample code (-1, -2, -3) represents the replicate number

To validate the reliability of DAPs identified in this work, the abundance of 5 DAPs involved in sugar metabolism were validated by western blot. Among them, 2 proteins, m.120,013 (FBA, fructose-bisphosphate aldolase) and m.97,819 (beta-glucosidase 30-like) identified both in this study and previous work, have also been validated by western blot in previous work [19]. The protein m.68,843 (phosphoenolpyruvate carboxylase) showed higher expression in CK than that in ethephon treatment in both high- and low-sugar genotypes but showed no difference between high- and low-sugar genotypes irrespective of treatment, indicating that m.68,843 was negatively affected byethephon not by genotype. While m.138,161 (phosphoglycerate kinase) and m.141,239 (phospho-glucomutase) were highly expressed in low-sugar genotype than in high-sugar clone, ethephon failed to induce them in both genotypes, suggesting that their expression is genotype-dependent. The results from western blot confirmed the DAPs detected by the new statistical analyses in this study, proving the value of the statistical approaches described here for identifying DAPs in sugarcane (Fig. 4).

### Protein-protein interaction network

**Fig. 5:**
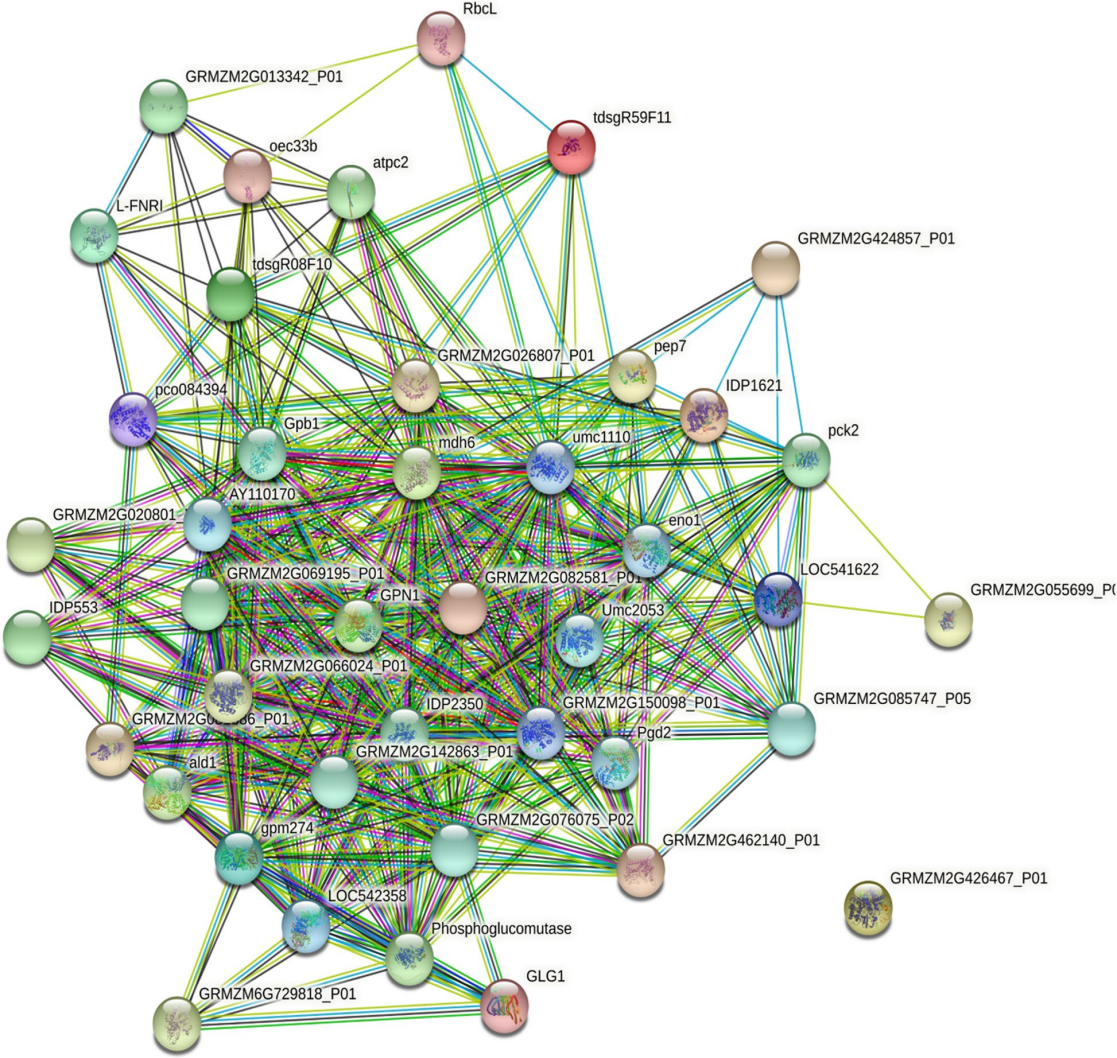
The protein-protein interaction network based on DAPs related to sugar metabolism. Each node represents a protein. The helical symbol in the node indicates the known 3D structure of the protein, and empty nodes indicate unknown proteins. The line between two nodes represents interaction and multiple lines represent various interactions between two proteins

Almost all the biological functions are mediated by proteins. Many proteins take part in biological processes such as signal transduction, gene expression, energy and nutrient metabolism by interaction with other proteins. PPI analysis predicts the function of proteins and identify key regulatory factors. To further explore the interaction between the 49 DAPs related to sugar metabolism identified in this work, a PPI network was constructed using STRING database. The PPI network contained 42 nodes and 350 edges. In the PPI network, 4 proteins (degree ≥ 26) were selected as hub proteins, including glucose-6-phosphate isomerase, 2-phospho-D-glycerate hydrolyase (enolase), malate dehydrogenase (NADP-dependent malic enzyme) and hosphor-glycerate kinase. Moreover, malate dehydrogenase had the highest degrees, suggesting that it may play a crucial role in sucrose accumulation in sugarcane (Fig. 5).

## Discussion

Sugarcane sucrose content improvement by conventional breeding programme is concerningly low due to the complex genetic background and slow introgression of exogenous genes [36]. To explore molecular breeding strategies for sugar content improvement, we are studying the molecular basis of sucrose accumulation in sugarcane [13, 16, 18, 19], with the ultimate aim of identifying potential molecular targets for variety improvement. Recently, we have developed a proteomic dataset developed for sucrose accumulation studies, and it is used in this study. After acquiring the raw sugarcane proteomics data, and conducting routine data pre-processing such as protein feature quantification, baseline correction and normalization, we conducted three different types of statistical analyses such as (i) principal component analysis (PCA), (ii) protein differential expression analysis and (iii) protein interaction network modelling, and they revealed biologically meaningful results from different perspectives. The PCA results (Fig. 1b) gave an overall picture of how the proteomics data of different samples varied. The first PC clearly separated M400 from the other three groups, indicating that when the sucrose content is low, the treatment of ethephon has a significant effect on protein abundance, which was also supported by our transcriptomic studies [18]. In that work, more DEGs were induced by ethephon in low-sugar genotypes compared to high-sugar clone [18]. On the other hand, the second PC separates the groups M400, and MCK from the other two groups R400, and RCk. Hence, the second PC explained the difference in protein expression between samples from the groups of low and high sucrose content, which was consistent with the finding that genotype had a dominant effect on gene expression than ethephon treatment [18].

We then evaluated and compared the three high dimensional statistical approaches, including the logistic marginal regression method [20], the logistic Elastic net method [21], and the logistic Bayesian stochastic search variable selection (SSVS) method [22], for detecting significantly differentially abundant proteins from this proteomics data comprising thousands of features that are associated with vital traits of interest. The marginal regression methods analyses one feature at a time, and then use a multiple testing procedure to eliminate the false positive finds. While the Elastic net and Bayesian approaches simultaneously analyse multiple protein features, and use penalty functions or prior information to conduct feature selection. The logistic Elastic net and Bayesian SSVS methods are closely related approaches and both of them analyse multiple features simultaneously and are able to conduct variable selection to select only a set of important features into the final model. However, the two approaches are philosophically different. The Elastic net, as a frequentist approach, relies on the likelihood model to make inference, and assumes all the model parameters to be fixed. But the Bayesian approach uses the posterior distribution (the likelihood combines with model prior) as a foundation to make inference, and all the model parameters have a marginal posterior distribution. Therefore, the uncertainty measures such as standard errors or credible intervals, or model inclusion probability in the Bayesian SSVS were directly controlled [37]. In the sugarcane proteomics dataset with 12 samples and about 3,000 protein features, the performance of the three methods on detecting different expressed features associated with either (i) high sugar VS low sugar content and (ii) water VS ethephon treatment was evaluated. The simple regression approach is the most liberal approach which detected 466 significant DAPs (Table S1; S5), accounting for 91.9% of total DAPs, even using the Bonferroni adjustment which is often considered as the most conservative approach for multiple testing. So, the simple regression is the most effective method for analysis of the proteomics dataset. However,, the multiple regression-based Bayesian SSVS detected 41 more significant DAPs, while the Elastic net method detected 40 and 60 DAPs from genotype and treatment comparisons, respectively (Table S2; S6),. All these DAPs were also detected by the simple regression analysis suggesting that the two approaches could confirm the analytical reliability of each other.

A total of 306 annotated DAPs were identified by the three statistical methods used in this research (Table S9), whereas only 139 DAPs were identified in our previous work using the same dataset [19]. By KEGG analysis and further comparison with our previous work, 42 new DAPs associated with sugar metabolism were identified in this work (Table 1). These DAPs provide new targets for further functional analysis and molecular breeding due to their potential biological functions in sugar metabolism. For instance, phospho-glucomutase (PGM) catalyzes the reversible conversion of glucose 1-phosphate (G1P) and glucose 6-phosphate (G6P), which is one of the vital enzymes promoting carbohydrate synthesis in higher plants [38, 39]. The lack of the cytosolic PGM activity in Arabidopsis resulted in decreased rosette fresh weight, shorter roots, and reduced seed production, leading to reduced growth [40]. The alpha-glucan phosphorylase is an important enzyme involved in carbohydrate metabolism in prokaryotes and eukaryotes. The plant alpha-glucan phosphorylase, also called starch phosphorylase, is known for the phosphorolytic degradation of starch, which is closely related to sugar metabolism [41]. In this work, 2 phospho-glucomutase (m.128,432/m.141,239) and one alpha-glucan phosphorylase (m.45,257) were newly detected, suggesting that they may play a crucial role in sugar metabolism.

The hub node in the PPT network may be the key regulatory factor in a pathway. The PPI analysis suggests that enzymes glucose-6-phosphate isomerase, malate dehydrogenase (NADP-dependent malic enzyme), enolase (2-phospho-D-glycerate hydrolyase) and phosphoglycerate kinase are hub proteins, with malate dehydrogenase scoring the highest degrees. So, these hub proteins may play a crucial role in sucrose accumulation in sugarcane. Glucose-6-phosphote isomerase catalyzes the inter-conversion of glucose 6-phosphate and fructose 6-phosphate, which plays an important role in sucrose synthesis. Over-expression of a wheat glucose-phosphate isomerase gene TaPGIc in *Arabidopsis thaliana* improved plant photosythesis, starch over-accumulation, biomass and yield [42].

Malate dehydrogenase catalyzes the reversible conversion of oxaloacetate and malate, and plays crucial roles in energy homeostasis and plant development [43]. Enolase (2-phospho-D-glycerate hydrolyase) exists in all eukaryotes and many prokaryotes. It converts 2-phospho-d-glycerate (PGA) into phosphoenolpyruvate (PEP) [44]. Mutation in AtENO2 (encodes an enolase) reduced seed starch level, resulting in decreased seed size and weight [45]. Phosphoglycerate kinase (PGK) is a key enzyme participating in both photosynthesis and glycolysis. In photosynthetic organisms, PGK catalyzes the reduction of 3-PGA to form sugar-phosphates [46, 47]. Malate dehydrogenase, enolase and PGK are three key enzymes in carbon fixation in photosynthetic organisms, so they are also closely associated with sugar metabolism.

**Fig. 6:**
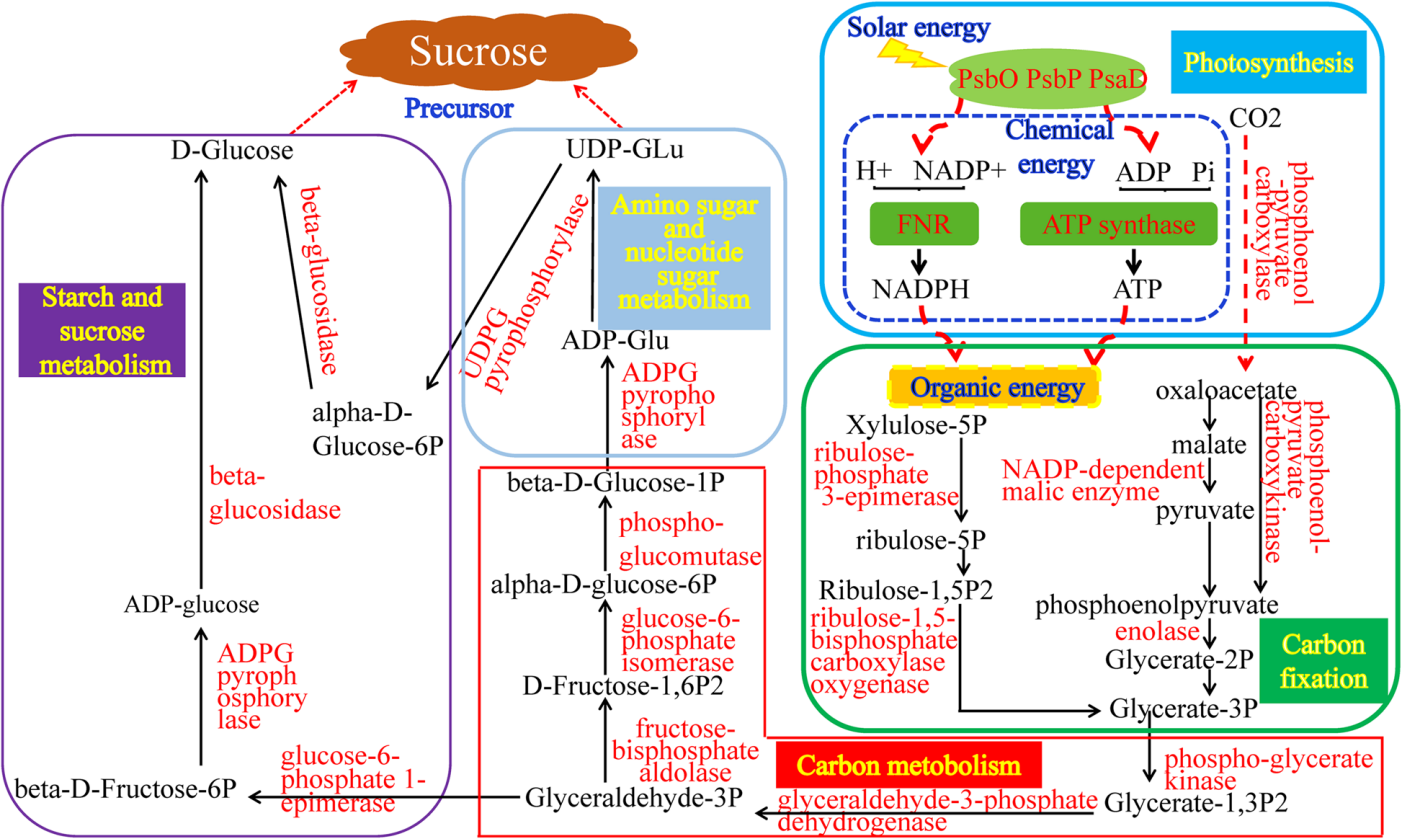
The putative network of key proteins associated with sucrose accumulation in sugarcane

Based on our work, a putative network of key proteins regulating sucrose accumulation is proposed in sugarcane (Fig. 6).

## Conclusions

This work, for the first time, used three logistic regression approaches including a classical marginal regression approach and two multiple regression methods to identify DAPs in a proteomics dataset. With this data analysis approach, more DAPs and more new proteins associated with sugar metabolism were identified, suggesting that this approach could be an additional method of proteomics data analysis in sugarcane. The proteins related to sugar metabolism form potential candidates for sucrose improvement in sugarcane by molecular breeding. The network of key proteins related to sugar metabolism based on the findings of this work provides a framework for dissecting the mechanism(s) of sucrose accumulation in sugarcane.

## Supplementary Information

**Additional file 1.**

**Additional file 2.**

**Additional file 3.**

**Additional file 4.**

**Additional file 5.**

**Additional file 6.**

**Additional file 7.**

**Additional file 8.**

**Additional file 9.**

**Additional file 10.**

**Additional file 11.**

**Additional file 12.**

## Data Availability

All the data supporting the findings of this study are available within the article and its supplementary materials. The mass spectrometry proteomics data have been deposited to the ProteomeXchange Consortium with the dataset identifier PXD024569 [19].

## References

[1] Emon JM (2016). The Omics Revolution in Agricultural Research. J Agric Food Chem.

[2] Li C, Jiang W, Xu Y (2017). Omics and Bioinformatics: Time for New Data Analysis Approaches?. OMICS..

[3] Demidenko E (2016). The p-Value You Can't Buy. Am Stat..

[4] Lualdi M, Fasano M (2019). Statistical analysis of proteomics data: A review on feature selection. J Proteomics.

[5] Meinshausen N, Bühlmann P (2006). High-dimensional graphs and variable selection with the Lasso. Ann Stat..

[6] Garcia-Milian, Hersey D, Vukmirovic M, Duprilot F (2018). Data challenges of biomedical researchers in the age of omics. Peer J.

[7] Moore PH (1995). Temporal and spatial regulation of sucrose accumulation in the sugarcane stem. Aust J Plant Physiol.

[8] FAOSTAT. FAOSTAT Crops and Livestock Products. Food and Agriculture Organization of the United Nations. 2020; http://www.fao.org/faostat/en/#data/QCL

[9] Oladosu Y, Rafii MY, Samuel C, Fatai A, Magaji U, Kareem I (2019). Drought resistance in rice from conventional to molecular Breeding: A review. Int J Mol Sci..

[10] Hagely KB, Jo H, Kim JH, Hudson KA, Bilyeu K (2020). Molecular-assisted breeding for improved carbohydrate profiles in soybean seed. Theor Appl Genet.

[11] Allwright MR, Taylor G (2016). Molecular Breeding for Improved Second Generation Bioenergy Crops. Trends Plant Sci.

[12] Wang A, Huang W, Niu J, Liu M, Yang L, Li Y (2013). Effects of ethephon on key enzymes of sucrose metabolism in relation to sucrose accumulation in sugarcane. Sugar Tech..

[13] Huang DL, Gao YJ, Gui YY, Chen ZL, Qin CX, Wang M (2016). Transcriptome of high sucrose sugarcane variety GT35. Sugar Tech..

[14] Huang DL, Qin CX, Gui YY, Zhao LH, Chen ZL, Wang M (2017). Role of the SPS gene families in the regulation of sucrose accumulation in sugarcane. Sugar Tech.

[15] Thirugnanasambandam PP, Hoang NV, Furtado A, Botha FC, Henry RJ (2017). Association of variation in the sugarcane transcriptome with sugar content. BMC Genom.

[16] Wang M, Li AM, Liao F, Qin CX, Chen ZL, Zhou L (2022). Control of sucrose accumulation in sugarcane (Saccharum spp. hybrids) involves miRNA-mediated regulation of genes and transcription factors associated with sugar metabolism. Glob Change Biol Bioenergy.

[17] Li YR, Solomon S, Ethephon (2003). A versatile growth regulator for sugar cane industry. Sugar Tech.

[18] Chen ZL, Qin CX, Wang M, Liao F, Liao Q, Liu XH (2019). Ethylene-mediated improvement in sucrose accumulation in ripening sugarcane involves increased sink strength. BMC Plant Biol.

[19] Qin CX, Chen ZL, Wang M, Li AM, Liao F, Li YR (2021). Identification of proteins and metabolic networks associated with sucrose accumulation in sugarcane (Saccharum spp. interspecific hybrids). J Plant Interact.

[20] Peter Bühlmann, Kalisch M, Meier L (2014). High-Dimensional Statistics with a view toward applications in biology. Annu Rev Stat Appl.

[21] Zou H, Hastie T (2005). Regularization and Variable Selection via the Elastic Net. J. R. Stat. Soc., Ser. B.

[22] O’Hara RB, Sillanpää MJ (2009). A review of Bayesian variable selection methods: what, how and which. Bayesian Anal.

[23] Polson NG, Scott JM, Windle J (2013). Bayesian inference for logistic models using Polya-Gamma latent variables. J Am Stat Assoc..

[24] Guan Y, Stephens M (2011). Bayesian variable selection regression for genome-wide association studies, and other large-scale problems. Ann Appl Stat.

[25] Scott JG, Berger JO (2010). Bayes and empirical-Bayes multiplicity adjustment in the variable-selection problem. Ann Stat.

[26] Efron B, Tibshirani R, Storey JD, Tusher V (2001). Empirical Bayes analysis of a microarray experiment. J Am Stat Assoc.

[27] Ventrucci M, Scott EM, Cocchi D (2011). Multiple testing on standardized mortality ratios: a Bayesian hierarchical model for FDR estimation. Biostatistics.

[28] Wadsworth WD, Argiento R, Guindani M, Galloway-Pena J, Shelburne SA, Vannucci M (2017). An integrative Bayesian Dirichlet-multinomial regression model for the analysis of taxonomic abundances in microbiome data. BMC Bioinform.

[29] Storey JD (2003). The positive false discovery rate: a Bayesian interpretation and the q-value. Ann Stat.

[30] Wen X. A unified view of false discovery rate control: reconciliation of Bayesian and Frequentist approaches. 2018; Available at https://arxiv.org/abs/1803.05284.

[31] Xie C, Mao X, Huang J, Ding Y, Wu J, Dong S (2011). KOBAS 2.0: a web server for annotation and identification of enriched pathways and diseases. Nucleic Acids Res.

[32] Kanehisa M, Goto SKEGG (2000). Kyoto Encyclopedia of Genes and Genomes. Nucleic Acids Res.

[33] Kanehisa M (2019). Toward understanding the origin and evolution of cellular organisms. Protein Sci.

[34] Kanehisa M, Furumichi M, Sato Y, Ishiguro-Watanabe M, Tanabe M (2021). KEGG: integrating viruses and cellular organisms. Nucleic Acids Res.

[35] Franceschini A, Simonovic M, Roth A, Mering CV, Szklarczyk D, Pletscherfrankild S (2013). STRING v9.1: Protein-protein interaction networks, with increased coverage and integration. Nucleic Acids Res.

[36] Hemaprabha G, Mohanraj K, Jackson PA, Lakshmanan P, Ali GS, Li AM (2022). Sugarcane Genetic Diversity and Major Germplasm Collections. Sugar Tech.

[37] Kyung M, Gill J, Ghosh M, Casella G (2010). Penalized Regression, Standard Errors, and Bayesian Lassos. Bayesian Anal.

[38] Davies EJ, Tetlow IJ, Bowsher CG, Emes MJ. Molecular and biochemical characterization of cytosolic phos*phoglucomutase* in wheat endosperm *(Triticum aestivum* L. cv. Axona). J Exp Bot. 2003;54(386):1351–60. 10.1093/jxb/erg151 12709481

[39] Uematsu K, Suzuki N, Iwamae T, Inui M, Yukawa H (2012). Expression of Arabidopsis plastidial phosphoglucomutase in tobacco stimulates photosynthetic carbon flow into starch synthesis. J Plant Physiol.

[40] Malinova I, Kunz HH, Alseekh S, Herbst K, Fernie AR, Gierth M (2014). Reduction of the cytosolic phosphoglucomutase in Arabidopsis reveals impact on plant growth, seed and root development, and carbohydrate partitioning. PLoS One..

[41] Rathore RS, Garg N, Garg S, Kumar A (2009). Starch phosphorylase: role in starch metabolism and biotechnological applications. Crit Rev Biotechnol.

[42] Gao F, Zhang H, Zhang W, Wang N, Zhang S, Chu C (2021). Engineering of the cytosolic form of phosphoglucose isomerase into chloroplasts improves plant photosynthesis and biomass. New Phytol.

[43] Ma B, Yuan Y, Gao M, Xing L, Li C, Li M (2018). Genome-wide Identification, Classification, Molecular Evolution and Expression Analysis of Malate Dehydrogenases in Apple. Int J Mol Sci..

[44] Straeten D, Rodrigues-Pousada RA, Van Goodman HM, Montagu M (1991). Plant enolase: gene structure, expression, and evolution. Plant Cell.

[45] Liu Z, Zheng L, Pu L, Ma X, Wang X, Wu Y (2020). Affects the Seed Size and Weight by Adjusting Cytokinin Content and Forming ENO2-bZIP75 Complex in Arabidopsis thaliana. Front Plant Sci.

[46] Martin W, Schnarrenberger C (1997). The evolution of the Calvin cycle from prokaryotic to eukaryotic chromosomes: a case study of functional redundancy in ancient pathways through endosymbiosis. Curr Genet.

[47] Massange-Sánchez JA, Casados-Vázquez LE, Juarez-Colunga S, Sawers RJH, Tiessen A (2020). The Phosphoglycerate Kinase (PGK) Gene Family of Maize (Zea mays var. B73). Plants (Basel).

